# Medical Statistics

**Published:** 1838-07

**Authors:** 


					MEDICAL STATISTICS.
Statistics of the Lunatic Asylum at Aversa, (Naples.) By Filippo Volpicella.
Proportion of the Sexes. The proportion of male inmates to the females is
usually about two to one: thus, in 1833, out of 640 insane, 429 were males and
211 females, who were thus distinguished:
Maniacs . . . Males, 73 Females, 50
Monomaniacs . . ... 207 ... 87
Fatuous . . .. ... 60 ... 40
Idiots ... ... 58 .. 23
Epileptic, with delirium ... 31 ... 11
A little less than half the insane are monomaniacs, and a fifth part maniacs; of
these, the chance of cure is greatest: there is but little hope of the cure of the
fatuous, still less of the idiots, and none at all of the epileptic.
Occupation. With regard to the occupations of the men, there were 35 priests,
9 monks and friars, 5 public officers, 45 soldiers, 7 tradesmen, 70 gentlemen
having property, 4 advocates, 2 schoolmasters, 6 copyists, 6 students, 10 sailors,
58 artisans, 124 peasants, 14 house-servants, 9 porters. Of the women, 2 were
nuns, 37 gentlewomen, 88 artisans, 71 peasants, 13 servants.
1838.] Medical Statistics. 239
Causes. As far as could be ascertained, these were as follows:
Physical Causes. Masturbation, male 3; blindness, male 1; venereal excess,
male 12; excessive drinking, male 27, female 12; starvation, male 4; insolation,
male 2; repelled eruptions, male 6; repelled lactation, female 2; suppressed and
irregular menstruation, 28; suppressed gonorrhoea, female 2; syphilis, male 1 ;
piles, male 4; gout, male 2; fever, male 3, female 1; hysteria, 2; encephalitis,
male 1; apoplexy, male 9; epilepsy, male 25, female 8; congenital idiocy, male 8,
female 2; hereditary madness, male 1; chronic headach, male 1.
Moral Causes. Natural insuperable sadness (tristezza), male 48, female 25;
wounded vanity, male 8, female 1; disappointed ambition, male 12, female 4;
regrets, male 10, female 2; rage, male 3, female 1; despair, male 1; discourage-
ment (avvilimento), male 2, female 1; fear, male 15, female 9; terror, male 13,
female 3; hatred, male 5; religious scruples, male 19, female 13; disappointed
hope, male 3, female 2; deluded hope, male 4, female 1; remorse, malel; infi-
delity, conjugal, male 4, female 1; jealousy, male 21, female 18; disappointed
love, male 28, female 21; nostalgia, male 5; excess in study, male I; excessive
labour, male 1; family quarrels, male 11; domestic anxieties, male 2; ruined
fortune, male 24; poverty, male 52, female 31; exalted imagination, male 6,
female 3; death of relatives, male 5, female 7; attendance on the insane, male 1;
depraved habits, male 4; sensuality, male 8, female 11.
The following is a Table of the per cent age of Cases, Cures, and Deaths, for
the two decennial periods between 1813 and 1832.
Jan. Feb. Mar. Apr.
Cases, 1 5ft 4ft 6ft
2 5to 7t$ 7to ^to
May
Cures, 1 7ft 2ft 6ft 5ft 5
^ ^13 *Oft ^ft
Deaths, 1 14ft 7ft ^ -t-j-g j ^10
2 13ft 12 7ft 7ft : lft
June July
"A I1 Oft
11A |12tc
'15
11TO
11
TO
3ft j 5ft
6-fc 7fo
Aug. Sep. Oct. Nov. Dec.
12ft 8ft 7ft 6ft 8
]0~ro 8to 810 5to 4ft
8 12ft 12ft 9 10ft
lOft 9ft 4ft
9-j5 7ft 9 12 lift
6ft ^ 6ft 8ft 10ft
The admissions, in the course of these twenty years, have been?men 2,775,
women 1,122: total 3,897. The readmissions, from relapses, were?men 22,
women 39; total 61, (131 in the paper.) There have escaped, of men 70, women
1 ; total 71. Relieved and intrusted to their friends, men 369, women 179;
total 548. Cured?men 1,072, women 442; total 1514. Dead?men 909,
women 313; total 1,222. The number admitted, and of those previously in the
hospital, are, to the cured, as 1 to 2^ of the males, and as 1 to 2^fg for the females:
to the dead, as 1 to 3^ for the males, and as 1 to 3^ for the females. [This is
evidently a mistake: it should be, the proportion of cures, &c. to admissions
and patients already in confinement; but, for want of the latter element of the
computation, or even an average of annual admissions, I cannot prove the cor-
rectness of the assertion.?Translator's note.']
The deaths for one year were?sanguineous apoplexy, males 14, females 8;
apoplexy supervening upon a violent epileptic attack, males 7? females 1; serous
apoplexy, females 5; aneurism of the heart, male 1, females 2; angina cesophagsea,
male 1; convulsive asthma, male 1, females 4; synochus, males 3, female 1; col-
liquative diarrhoea, males-8, females 2; dysentery, males 7, females 11; nervous
phthisis, males 9, (perhaps marasmus is meant;) phthisis pulmonalis, males 10,
females 3; phthisis mesenterica, males 8, females 7; intestinal phthisis, (probably
ulceration of Peyer's glands,) female 1; haemoptysis, male 1; gangrene from
lying, male 7; pneumonia, males 2, females 2; hydrothorax, male 1, female 1;
ascites, male 1, female 1; anasarca, males 3, females 3. Total males 84, females
52. Filiatre Sebezio. 1836.
240 Selections from the Foreign Journals. [J uly,
Statistics of Insanity in Westphalia and Saocony.
The number of cases of congenital idiocy in the Prussian province of
Westphalia amounted, in 1834, to 454 males and 274 females; together to 728:
equal to 47 per cent, of the total number of cases of insanity in the province,
which amounted to 1,535, or to one in 846 of the population. Of this number
507 were males, giving one case for every 712 male inhabitants, and 339 females,
or one for every 1010 of the female population. Classed according to religious
denomination, there was one case for every 962 of the members of the re-
formed church, one for every 786 of the Roman catholic population, and one for
every 758 of the Jewish population.
In the province of Saxony there were, in 1836, 1,580 insane in a population of
1,529,607, or one in 968. Of this number 882 were males, and 698 females:
namely, unmarried, males 732, females 526; married, males 114, females 86;
widowers 36, widows 86. Classed according to age, the numbers were?under
fifteen years of age, 85 males and 57 females; between fifteen and thirty, 286
males and 212 females; between thirty and forty-five, 289 males and 226 females;
between forty-five and sixty, 177 males and 147 females; and, lastly, above sixty
years of age, forty-five males and fifty-six females.
It is a matter of extreme difficulty to decide with certainty upon the cause of the
disease in a vast number of cases, but it often happens that a probable cause may
be assigned. In this way, corporeal causes were assumed in 196 males and 137
females; mental causes in 127 males and 114 females; hereditary predisposition
in 119 males and 66 females; and, lastly, no probable cause could be assigned in
440 males and 381 females. Under the head of dementia were classed 657 males
and 485 females; under that of monomania, 178 males and 177 females; and un-
der that of mania, 47 males and 36 females. The first class comprehended 118
epileptics, the second 32, and the third 12. Of the total number, 597 belonged to
towns, and 983 to the country.
Medicinische Zeitung. No. 47. 1837.
STATISTICAL NOTICE OF THE CHOLERA-EPIDEMIC IN BERLIN, IN THE YEAR 1837.
BY DR.CASPER.
Weeks. Sick. Dead.
1. August 12-18 . . 117 .. 65
2. 19-25 . . 575 .. 305
3. 26, September 1 . 693 .. 395
4. September 2-8 . . 573 .. 335
5. 9-15 . 486 .. 331
6. 16-22 . . 513 .. 289
7. 23-29 . . 254 .. 181
8. 30, October 6 . 128 .. 120
9. October 7-13 . . 65 .. 42
10. 14-20 . . 59 .. 37
11. 21-27 . . 44 .. 32
12. 28, November 3 . 29 .. 18
13. November 4-10 . . 15 .. 16
14. 11-17 . 6 .. 5
15. 18-24 . . 3 .. 1
16. 25 . 1 .. 2
Total
:?561 2174
The cholera-epidemic of 1831 prevailed, in Berlin, during a period of forty-six days
longer than that of 1837. The numbers, in 1831, were?taken ill, 2271; died, 1426;
being less than in 1837 by 1290 and 748 respectively, and making the proportion of
deaths to the sick?in 1831, 0.372: 0.628; in 1837, 0.391: 609.
IVochenschrift f. d. ges. Heilkunde. Feb. 10, 1838.
1838.] Forensic Medicine. 241
STATISTICS OF SUICIDE IN LONDON. BY M. MOREAU DE JONNiS.*
I. Number of Deaths from Violence, in general.
1824. 828. 1829. 1830. 1831.
Suicide . 52 41 35 25 48
Executed 10 1 26 7 6
Murdered ..2 6 4 2 5
Poisoned . 4 27 7 4 7
Found dead . 5 15 6 13 5
Drowned . 149 150 36 97 131
Burnt . 36 47 53 61 35
From famine . 110 0 1
From excessive drinking 5 7 3 4 0
From suffocation . 5 10 10 5 5
II. Number of Suicides in London, during a century and half.
From 1690 to 1699 . 236
1700?1709 . 278
1710 ? 1719 . 301
1720 ? 1729 . 478
1730?1739 . 501
1740 ? 1749 . 422
1750 ? 1759 . 363
From 1760 to 1769 . 351
1770 ? 1779 . 339
1780 ? 1789 . 224
1790 ? 1799 . 274
1800 ? 1809 . 347
1810 ?1819 . 362
1820 ?1829 . 381
hi. Number and Proportion of Suicides in the chief Capitals of Eur ope. f
Berlin . . 1813?1822 360
Copenhagen . 1804?1806 100
Naples . . 1828 330
Hamburg . . 1822 59
Berlin . . 1799?1808 60
Paris . ? 1836 341
Milan . .1827 37
Berlin . . 1788?1797 35
Vienna . ? 1829 45
Prague . ? 1820 6
Petersburgh . . 1831 22
London . . 1834 42
Naples . 1826 13
Palermo . . 1831 2
Periods. Suicides. Proportion to Population.
i 750
? 1,000
? 1,100
? 1,800
? 2,300
? 2,700
? 3,200
? 4,500
? 6,400
? 16,000
? 21,000
? 21,000
? 27,000
? 173,000
" From this it appears," says M. M. de Jonnbs, "that the inhabitants of London
are much less inclined to suicide than those of most of the cities of Europe: conse-
quently, that the notion that the climate of England predisposes to suicide is altoge-
ther erroneous." Gazette M6d. de Paris. 24 Feb. 1838.
* From an unpublished work on the Population of London.
t Compare this with the tables given at pp. 371-2 in the last Number.?Ed.

				

